# Prevalence, associated factors, and gene polymorphisms of obesity in Tibetan adults in Qinghai, China

**DOI:** 10.1186/s12889-023-17181-7

**Published:** 2024-01-26

**Authors:** Ye Wang, Li Pan, Huijing He, Zhanquan Li, Sen Cui, Airong Yang, Wenfang Li, Guoqiang Jia, Ximing Han, Xianghua Wang, Guangliang Shan

**Affiliations:** 1 School of Population Medicine and Public Health, Chinese Academy of Medical Sciences and Peking Union Medical College, Beijing, China; 2 Department of Epidemiology and Statistics, Institute of Basic Medical Sciences, School of Basic Medicine, Chinese Academy of Medical Sciences, Peking Union Medical College, 5 Dong Dan San Tiao, Dong Cheng District, Beijing, 100005 China; 3 Qinghai University Affiliated Hospital, Qinghai, China; 4 Institute of Biomedical Engineering, Chinese Academy of Medical Sciences, Peking Union Medical College, 236 Baidi Road, Nankai District, Tianjin, 300192 China

**Keywords:** Tibetan, Obesity, Prevalence, Genetic epidemiology, Environmental factors

## Abstract

**Objectives:**

To explore the prevalence and associated factors of obesity in Tibetan adults in Qinghai, China, and to determine the association between the *FTO* (rs1121980 and rs17817449) and *MC4R* gene (rs17782313 and rs12970134) polymorphisms with obesity.

**Methods:**

A cross-sectional survey was conducted in 2015 in Qinghai to selected Tibetan adults aged 20 to 80 years. Prevalence of obesity (BMI ≥ 28 kg/m^2^) and overweight (BMI 24 ~ 27.9 kg/m^2^) were evaluated. Multivariable logistic models were used to determine the associated factors. Pair-matched subjects of obesity cases and normal-weight controls were selected for the gene polymorphism analyses. Conditional logistic models were used to assess the association between gene polymorphisms with obesity. Additive and multiplicative gene-environment interactions were tested.

**Results:**

A total of 1741 Tibetan adults were enrolled. The age- and sex- standardized prevalence of obesity and overweight was 18.09% and 31.71%, respectively. Male sex, older age, heavy level of leisure-time exercise, current smoke, and heavy level of occupational physical activity were associated with both obesity and overweight. *MC4R* gene polymorphisms were associated with obesity in Tibetan adults. No significant gene-environment interaction was detected.

**Conclusion:**

The prevalence of obesity and overweight in Tibetan adults was high. Both environmental and genetic factors contributed to the obesity prevalent.

**Supplementary Information:**

The online version contains supplementary material available at 10.1186/s12889-023-17181-7.

## Introduction

Obesity is one of the fast-growing health problems worldwide. The prevalence of obesity is rapidly rising in both developed and developing countries [1, 2]. Obesity is a widely-recognized risk factor for cardiovascular disease, diabetes mellitus, chronic kidney disease, and some types of cancer [3–6]. The high prevalence of obesity has led to a huge burden of obesity-related illnesses [7].

In China, the prevalence of obesity greatly increased over the past decades [8]. The up-to-date data reported that half of the adults in China were prevalent of overweight or obesity [9]. The obesity pandemic is greatly influenced by the combination of physiological and behavioral factors that are triggered by the changes in the food environment and the environmentally driven reductions in physical activity [10]. China is a multi-ethnic nation, with 56 distinct ethnicities living in the vast territory. The prevalence of obesity among different ethnicities differs due to the disparities in lifestyle, genetics, living environment, socioeconomics, etc [11, 12]. Previous publications have reported the differences in the prevalence of cardiovascular disease risk factors including obesity [13–16].

Tibetan is one of the largest ethnic minorities in China, mainly living in the Qinghai-Tibet Plateau with an average altitude higher than 3000 m. Many Tibetans live as pastoralists on the plateau and keep a relatively primitive lifestyle. The natural environment in the plateau, socioeconomic condition, unique lifestyles, and genetic features may significantly impact the obesity epidemic. However, few studies in China evaluate the prevalence and the determinants of obesity in Tibetan adults. The substantial contribution of genetic factors to obesity has been well-established. The *FTO* was the first GWAS-identified obesity gene in 2007 [17]. Before GWAS, MC4R deficiency was identified as the commonest monogenic form of obesity [18]. The *FTO* and *MC4R* gene are two of the verified obesity-related variants, but evidence from ethnic minorities in China is limited. Especially, in Tibetan adults, the role of the *FTO* and *MC4R* gene and their interaction with environmental factors is unclear. The China National Health Survey (CNHS) [19] provides the opportunity to compare not only the health status but also the genetic backgrounds of different ethnic groups. It is essential to determine the factors related to obesity of different ethnic groups in China.

In this study, we used cross-sectional data to assess the prevalence and associated factors of obesity in Tibetan adults living in Qinghai Province in China. Based on the surveyed population, a 1:1 pair-matched case-control study was designed to determine the association between *FTO* (rs1121980 and rs17817449) and *MC4R* gene (rs17782313 and rs12970134) polymorphisms with obesity in Tibetan adults. The gene-environment interaction was also tested.

## Methods

### Study population

This study was one part of the (CNHS), which was a nationwide multi-ethnic cross-sectional study conducted from 2012 to 2017 in eleven provinces in mainland China. The CNHS was designed to obtain the reference intervals of physiological constants and the determinants of noncommunicable diseases among different ethnic populations aged 20 to 80 years in China. Details of the sampling procedures have been described elsewhere [19]. The eligibility criteria were as follows: (1) aged 20–80 years, (2) living in their current residence for at least 1 year, and (3) the participant and his/her parents were all Tibetan. The exclusion criteria included: (1) individual with severe mental or physical illness, (2) pregnant females, and (3) military personnel on active service. In this present study, a stratified cluster sampling method was used to select Tibetan in Qinghai province in 2015. In the first stage, to account for socioeconomic development and distribution of ethnic residence, Xining City (the capital of Qinghai Province), Hainan Tibetan Autonomous Prefecture, and Haidong City were selected. In the second stage, Chengxi District in Xining, Gonghe County and Guide County in Hainan, and Hualong County in Haidong were selected. In the third stage, Qiabuqia Town and Jiangxigou Town in Gonghe, Heyin Town in Guide, and Bayan Town in Hualong were selected as the survey sites. In the final stage, adults aged 20 to 80 years and resident in the selected sites for at least 1 year before the survey were eligible to participate (n = 1741). To account for genetic ancestry, we identified Tibetan people as individuals whose parents were both of Tibetan ethnicity. All participants provided written informed consent before the survey. The study was approved by the bioethical committee of Institute of Basic Medical Sciences, Chinese Academy of Medical Sciences, Beijing, China.

**Fig. 1:**
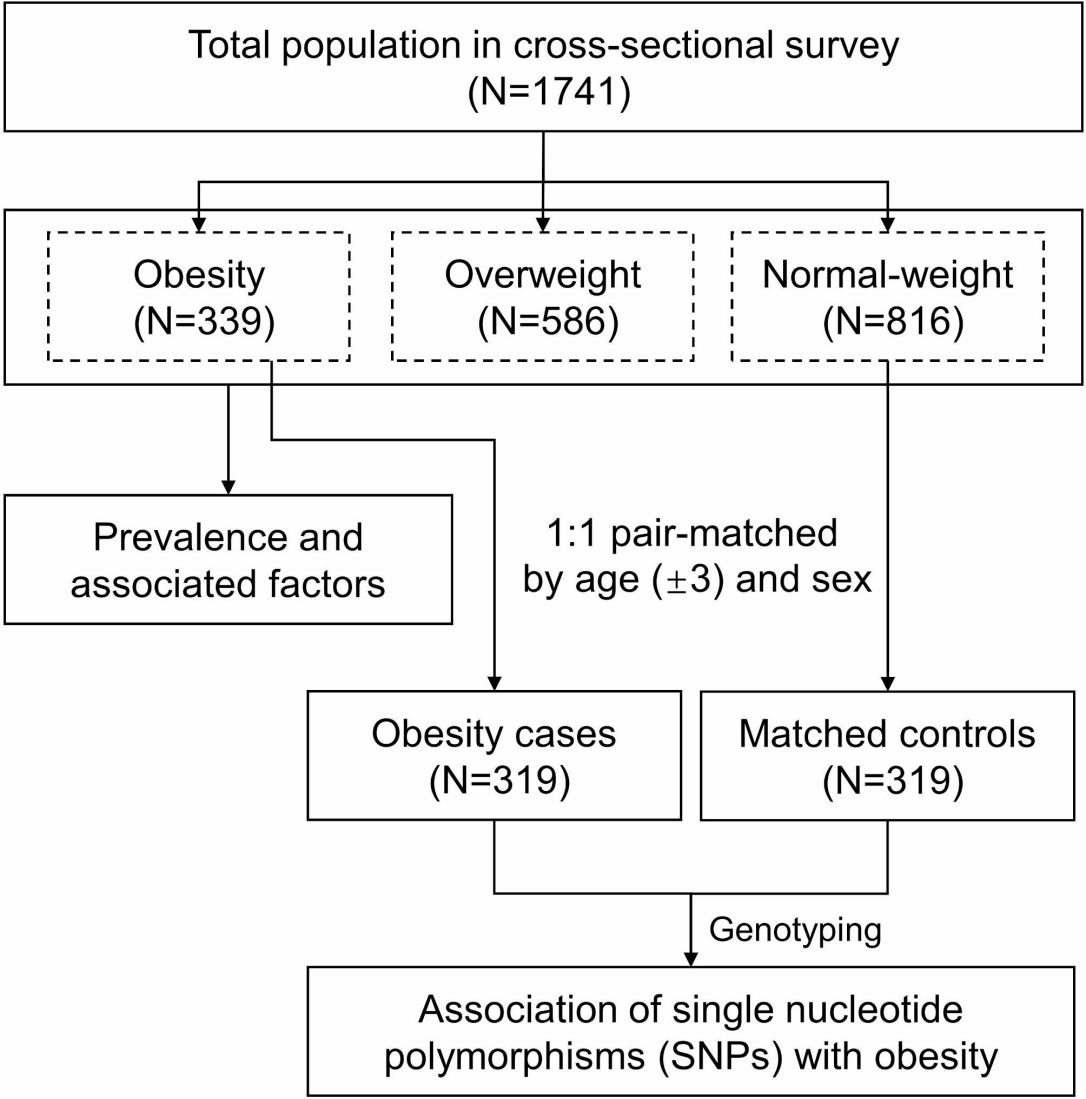
The flowchart for selecting participants in this study

Based on the cross-sectional survey, a 1:1 pair-matched case-control study (matched for age (± 3 years) and sex) was then designed for the SNPs analyses. Those with BMI ≥ 28 kg/m^2^ were all included in the case group (n = 319), and their counterparts with BMI < 24 kg/m^2^ were eligible for the control group selection (n = 319). The flowchart for the study population enrollment is shown in Fig. 1.

### Data collection

In the fieldwork, a standardized questionnaire was used to collect social-demographic characteristics (sex, age, ethnicity, education, income, etc.) and lifestyles (smoking, drinking, physical activity, etc.) through face-to-face interviews. The interviews were conducted by well-trained medical staff.

Education was categorized into three categories according to the last educational institution attended: primary school or below, junior or senior high school, and college or above. Personal annual income was categorized into ≤ 10,000 and > 10,000 CNY according to the median. Current smoking was defined as smoking at least one cigarette per day for at least 6 months. Current drinking was defined as drinking at least twice per month with an intake of more than 640 ml (one bottle) of beer or 100 ml of liquor for at least 6 months. Occupational physical activity was grouped into light, moderate, and heavy according to intensity. The leisure-time exercise was considered participation in moderate or vigorous activity for at least 20 min at leisure time, with three levels: light (< 1 day per week), moderate (1–4 days per week), and heavy (5–7 days per week).

The body weight was measured in light clothing using a body composition analyzer (BC-420, TANITA, Japan) with an accuracy of 0.1 kg. Height was measured with bare feet by a fixed stadiometer with an accuracy of 0.1 cm. The average of two height measurements was recorded. The body mass index (BMI) was calculated as the weight in kilograms divided by the square of the height in meters.

### Genotyping

The genomic DNA was extracted from blood samples using the magnetic bead-based kit. The *MC4R* rs17782313 (T > C), rs12970134 (G > A), *FTO* rs1121980 (G > A), and rs17817449 (T > G) polymorphisms were genotyped using TaqMan assay. For the four SNPs, the PCR reaction was optimized in a 10 µL total volume containing 1 µL DNA template, 5 µL Master Mix, 3 µL primer, and 3 µL water.

### Definition

According to the diagnostic criteria for the Chinese population by the Working Group on Obesity in China (WGOC) [20], obesity was defined as BMI ≥ 28 kg/m^2^, and overweight was defined as 24 ≤ BMI < 28 kg/m^2^. Participants with 18.5 ≤ BMI < 24 kg/m^2^ were defined as normal-weigh.

### Statistical analysis

Descriptive results were stratified by BMI categories. Data were summarized as numbers (percentage) for categorical variables and mean ± SD for continuous variables. The differences between groups were compared using chi-square tests or analysis of variance.

The standardized prevalence of obesity and overweight was calculated based on the distributions of the 2010 China census population using the direct method. Multivariable logistic regression models that took account of the complexity of the sampling strategy were applied to determine the associated factors of obesity and overweight, using sociodemographic and lifestyle factors as the independent variables [21]. As a sensitive analysis, participants with BMI < 18.5 kg/m^2^ were deleted to determine the associated factors.

In the case-control analyses, the Hardy-Weinberg equilibrium (HWE) was tested to assess the deviation between observed and expected genotype frequencies using chi-square tests in the control group. Chi-square tests were used to compare genotypes and allele frequencies between the obesity cases and the controls.

To determine the association between *MC4R* and *FTO* gene polymorphisms with obesity, conditional logistic regression models were applied to test all four inheritance models for each variant (dominant, recessive, codominant, and additive). All models were adjusted by socioeconomic status and lifestyle factors. The unweighted genetic risk score (GRS) was then derived by summing the total number of risk alleles for each individual across the four SNPs [22]. To account for uncertainty in risk allele effect sizes, the weighted genetic risk score (wGRS) was calculated as the sum of the number of risk alleles multiplied by the corresponding log odds ratio of each risk allele [23]. To assess the association between GRS and wGRS with obesity, the sample was categorized into three groups according to tertiles.

Both multiplicative and additive interactions were evaluated to examine the interaction between SNPs and behavioral factors (smoking, alcohol drinking, occupational physical activity, and leisure-time exercise in this study) on obesity. The multiplicative interactions were evaluated by the product terms added in the regression models. The relative excess risk due to interaction (RERI), attributable proportion due to interaction (AP), and synergy index (SI) were used to evaluate the additive interactions, which were calculated using the regression coefficients and covariance matrix. The 95% confidence intervals (CIs) of RERI, AP, and SI were calculated by the delta method [24]. If the 95% CIs of RERI and AP did not include 0 and the 95% CI of SI did not include 1, an interaction was present.

All analyses were conducted using SAS statistical software (version 9.4; SAS Institute Inc., Cary, NC, USA). A 2-tailed alpha with *P* < 0.05 was considered statistically significant.

## Results

A total of 1,741 Tibetan adults were included in the cross-sectional survey, half of whom were male participants (870/1741). The characteristics of the participants are summarized in Table 1. The mean age of the study population was 43.96 ± 12.55 years. Compared to normal-weight, the subjects with overweight and obesity were at an older age and had higher proportions of male participants. Details of the comparison in demographic characteristics are displayed in Table 1.

**Table 1:** Basic characteristics of study sample by BMI categories

		Total (N = 1741)	Normal-weight (N = 816)	Overweight (N = 586)	Obesity (N = 339)	*P* ^*a*^	*P* ^*b*^
Sex, n (%)						< 0.0001	< 0.0001
	Males	870 (49.97)	356 (43.63)	322 (54.95)	192 (56.64)		
	Females	871 (50.03)	460 (56.37)	264 (45.05)	147 (43.36)		
Age (years)		43.96 ± 12.55	40.78 ± 13.19	45.80 ± 11.25	48.44 ± 11.04	< 0.0001	< 0.0001
Age (years), n (%)						< 0.0001	< 0.0001
	20 ~ 29	258 (14.82)	198 (24.26)	47 (8.02)	13 (3.84)		
	30 ~ 39	386 (22.17)	204 (25.00)	126 (21.50)	56 (16.52)		
	40 ~ 49	517 (29.70)	206 (25.25)	194 (33.11)	117 (34.51)		
	50 ~ 59	366 (21.02)	120 (14.71)	151 (25.77)	95 (28.02)		
	60 ~ 80	214 (12.29)	88 (10.78)	68 (11.60)	58 (17.11)		
Residence						0.4591	0.0425
	Urban	843 (48.42)	400 (49.02)	299 (51.02)	144 (42.48)		
	Rural	898 (51.58)	416 (50.98)	287 (48.98)	195 (57.52)		
Income (CNY/y), n (%)						0.4458	0.0026
	< 10,000	853 (48.99)	390 (47.79)	268 (45.73)	195 (57.52)		
	≥ 10,000	888 (51.01)	426 (52.21)	318 (54.27)	144 (42.48)		
Education						0.4868	0.0056
	Primary or below	1,007 (57.84)	448 (54.90)	339 (57.85)	220 (64.90)		
	Junior or senior high	331 (19.01)	161 (19.73)	113 (19.28)	57 (16.81)		
	College or above	403 (23.15)	207 (25.37)	134 (22.87)	62 (18.29)		
Smoking status						0.0182	0.0007
	Never	1,260 (72.37)	605 (74.14)	406 (69.28)	249 (73.46)		
	Former	179 (10.28)	62 (7.60)	70 (11.95)	47 (13.86)		
	Current	302 (17.35)	149 (18.26)	110 (18.77)	43 (12.68)		
Drinking status						0.0061	0.1234
	Never	1,185 (68.06)	576 (70.59)	376 (64.17)	233 (68.74)		
	Former	196 (11.26)	72 (8.82)	81 (13.82)	43 (12.68)		
	Current	360 (20.68)	168 (20.59)	129 (22.01)	63 (18.58)		
Occupational physical activity, n (%)						0.0831	0.3480
	Light	1,241 (71.32)	561 (68.84)	434 (74.06)	246 (72.57)		
	Moderate	186 (10.69)	90 (11.04)	59 (10.07)	37 (10.91)		
	Heavy	313 (17.99)	164 (20.12)	93 (15.87)	56 (16.52)		
Leisure-time exercise, n (%)						< 0.0001	< 0.0001
	Light	705 (40.61)	379 (46.56)	213 (36.54)	113 (33.34)		
	Moderate	448 (25.81)	216 (26.54)	139 (23.84)	93 (27.43)		
	Heavy	583 (33.58)	219 (26.90)	231 (39.62)	133 (39.23)		

a: comparison between overweight and normal-weight; b: comparison between obesity and normal-weight

**Fig. 2:**
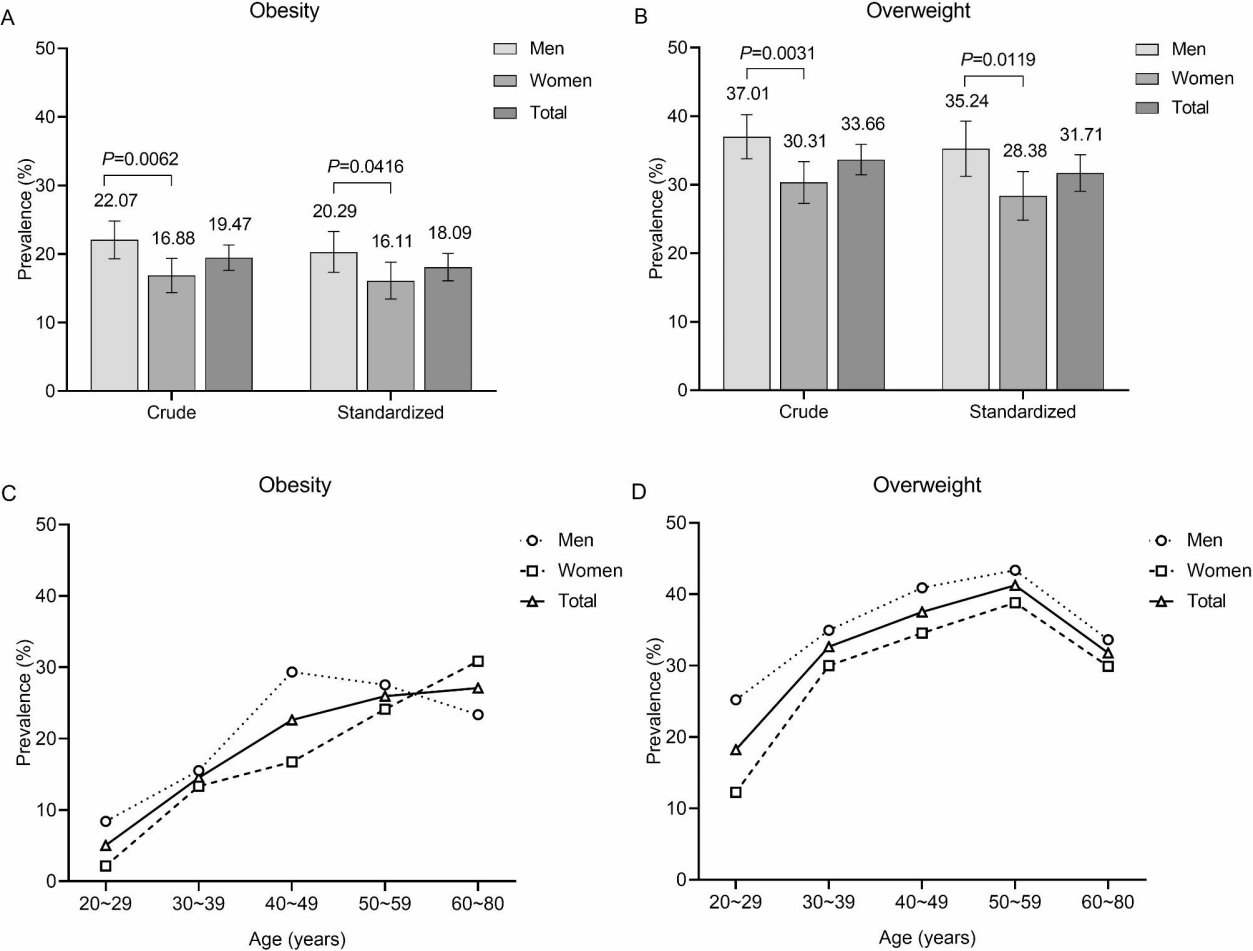
Sex- and age-specific prevalence of obesity and overweight in Tibetan adults

The crude and standardized prevalence of obesity and overweight are illustrated in Fig. 2A and B. The overall prevalence of obesity and overweight in Tibetan adults was 19.47% and 33.66%. Males had higher prevalence than females (22.07% vs. 16.88%, *P* = 0.0062; 37.01% vs. 30.31%, *P* = 0.0031). With standardized by age and sex, the prevalence of obesity and overweight was 18.09% and 31.71%. The age-standardized prevalence in males remained significantly higher than in females (20.29% vs. 16.11%, *P* = 0.0416; 35.24% vs. 28.38%, *P* = 0.0119). The age-specific prevalence of obesity in females increased with age, while in males the prevalence peaked at 40–49 years, and decreased. The age-specific prevalence of overweight in both males and females increased with age and peaked at 50–59 years (see Fig. 2C and D).

**Fig. 3:**
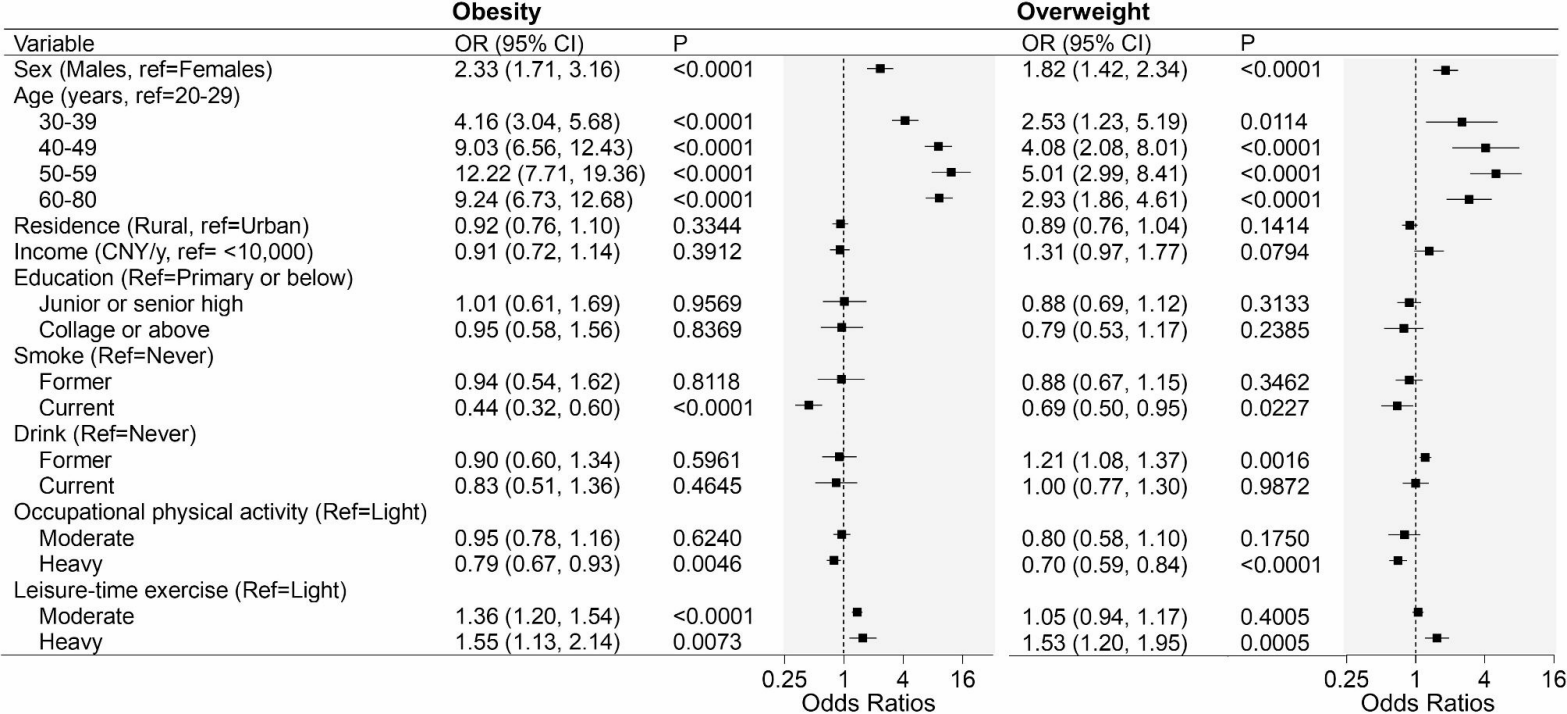
Multivariable logistic analysis of obesity and overweight in Tibetan adults

In multivariable analyses, male sex, older age, and heavy level of leisure-time exercise were found to be associated with higher odds of both obesity and overweight, while current smoke and heavy level of occupational physical activity were associated with lower odds. Relative to females, males were at 2.33-folds (95% CI: 1.71 to 3.16) and 1.82-folds (95% CI: 1.42 to 2.34) higher odds of obesity and overweight, respectively. Individuals aged 50–59 years were at the highest odds of obesity (12.22, 95% CI: 7.71 to 19.36) and overweight (5.01, 95% CI: 2.99 to 8.41) than the young adults (20–29 years). Those engaging in heavy leisure-time exercise were associated with 1.55-folds (95% CI: 1.13 to 2.14) and 1.53-folds (95% CI: 1.20 to 1.95) higher odds of obesity and overweight than those slightly engaging in. Current smoking was negatively associated with obesity (0.44, 95%CI: 0.32 ~ 0.60) and overweight (0.69, 95%CI: 0.50 ~ 0.95). Heavy level of occupational was found to be related to a lower risk of obesity (0.79, 95%CI: 0.67 ~ 0.93) and overweight (0.70, 95%CI: 0.59 ~ 0.84). (Fig. 3) The sensitive analysis with deleting participants with BMI < 18.5 kg/m^2^ showed consistent results (see supplementary Table S1).

In the case-control study, a comparable control group (n = 319) was selected for the cases (n = 319). As Table 2 shows, there was no significant difference between the cases and controls except for smoke and leisure-time exercise. The genotype and allele frequencies of the four SNPs in the case and control groups were not significantly different (*P* > 0.05 for all). The HWE tests for all four SNPs show genetic equilibrium (*P* > 0.05 for all, see Table 3).

**Table 2:** Basic characteristics of obesity cases and normal-weight controls in the case-control sample

		Case (N = 319)	Control (N = 319)	*P*
Sex, n (%)				0.9999
	Males	180 (56.43)	180 (56.43)	
	Females	139 (43.57)	139 (43.57)	
Age (years), n (%)				0.5711
	20 ~ 29	13 (4.08)	13 (4.08)	
	30 ~ 39	56 (17.55)	53 (16.62)	
	40 ~ 49	113 (35.42)	99 (31.03)	
	50 ~ 59	85 (26.65)	87 (27.27)	
	60 ~ 80	52 (16.30)	67 (21.00)	
Residence				0.4681
	Urban	134 (42.01)	125 (39.18)	
	Rural	185 (57.99)	194 (60.82)	
Income (CNY/y), n (%)				0.5757
	< 10,000	185 (57.99)	178 (55.80)	
	≥ 10,000	134 (42.01)	141 (44.20)	
Education				0.5806
	Primary or below	209 (65.52)	221 (69.28)	
	Junior or senior high	52 (16.30)	48 (15.05)	
	Collage or above	58 (18.18)	50 (15.67)	
Smoking status				< 0.0001
	Never	236 (73.98)	190 (59.56)	
	Former	44 (13.79)	43 (13.48)	
	Current	39 (12.23)	86 (26.96)	
Drinking status				0.0671
	Never	221 (69.28)	193 (60.50)	
	Former	38 (11.91)	48 (15.05)	
	Current	60 (18.81)	78 (24.45)	
Occupational physical activity, n (%)				0.1896
	Light	230 (72.10)	209 (65.51)	
	Moderate	36 (11.29)	42 (13.17)	
	Heavy	53 (16.61)	68 (21.32)	
Leisure-time exercise, n (%)				0.0005
	Light	101 (31.66)	149 (46.71)	
	Moderate	90 (28.21)	69 (21.63)	
	Heavy	128 (40.13)	101 (31.66)	

**Table 3:** Comparison of genotype and allele frequencies and the HWE test between case and control groups in Tibetan adults

	Case (N = 319)	Control (N = 319)	*P*	HWE-*P*
MC4R rs17782313				
Genotype			0.2157	0.9091
T/T	165 (51.73)	187 (58.62)		
T/C	133 (41.69)	114 (35.74)		
C/C	21 (6.58)	18 (5.64)		
Allele			0.1082	
T	463 (72.57)	488 (76.49)		
C	175 (27.43)	150 (23.51)		
MC4R rs12970134				
Genotype			0.2661	0.7177
G/G	182 (57.05)	202 (63.32)		
G/A	122 (38.25)	105 (32.92)		
A/A	15 (4.70)	12 (3.76)		
Allele			0.1202	
G	486 (76.18)	509 (79.78)		
A	152 (23.82)	129 (20.22)		
FTO rs1121980				
Genotype			0.7036	0.2614
G/G	187 (58.62)	194 (60.81)		
G/A	107 (33.54)	105 (32.92)		
A/A	25 (7.84)	20 (6.27)		
Allele			0.4293	
G	481 (75.39)	493 (77.27)		
A	157 (24.61)	145 (22.73)		
FTO rs17817449				
Genotype			0.7031	0.4469
T/T	194 (60.82)	199 (62.38)		
T/G	103 (32.29)	103 (32.29)		
G/G	22 (6.89)	17 (5.33)		
Allele			0.5010	
T	491 (76.96)	501 (78.53)		
G	147 (23.04)	137 (21.47)		

HWE: Hardy-Weinberg Equilibrium

**Fig. 4:**
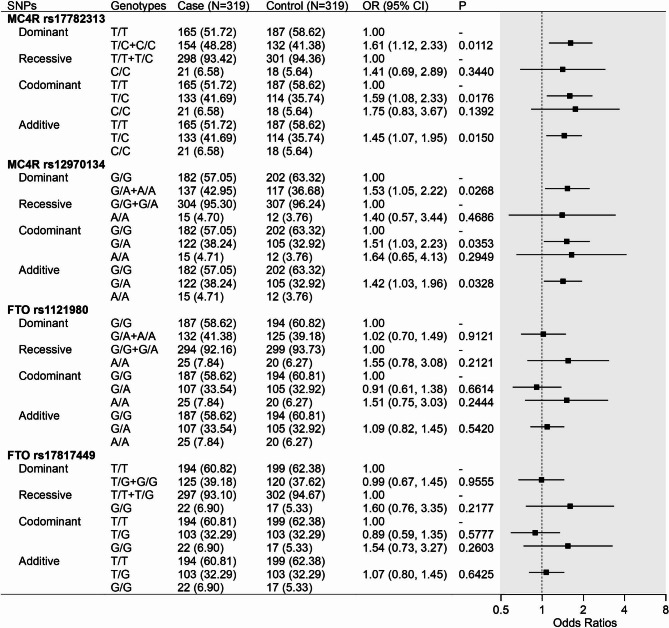
Association of FTO and MC4R gene polymorphisms with obesity in Tibetan adults Models were adjusted for residence, education, income, smoking status, drinking status, occupational physical activity, and leisure-time exercise

**Fig. 5:**
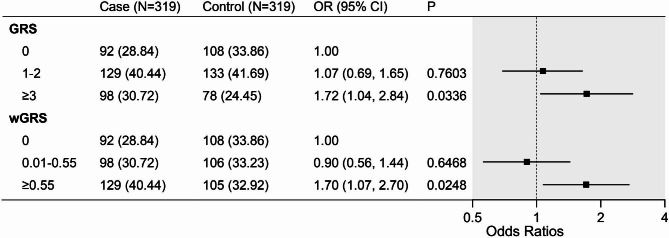
Association of genetic risk score (GRS) and weighted-genetic risk score (wGRS) with obesity in Tibetan adults Models were adjusted for residence, education, income, smoking status, drinking status, occupational physical activity, and leisure-time exercise

Figure 4 illustrates the association between *MC4R* and *FTO* gene polymorphisms with obesity in Tibetan adults by conditional logistic regression models. With adjustment for socioeconomic status and lifestyle factors, *MC4R* gene polymorphisms were significantly associated with obesity under dominant, codominant, and additive inheritance models (Rs17782313 genotype T/C + C/C vs. T/T OR = 1.61, 95%CI: 1.12 ~ 2.33; genotype T/C vs. T/T OR = 1.59, 95%CI: 1.08 ~ 2.33; additive model OR = 1.45, 95%CI: 1.07 ~ 1.95. Rs12970134 genotype G/A + A/A vs. G/G OR = 1.53, 95%CI: 1.05 ~ 2.22; genotype G/A vs. G/G OR = 1.51, 95%CI: 1.03 ~ 2.23; additive model OR = 1.42, 95%CI: 1.03 ~ 1.96). The SNPs of *FTO* were not found to be significantly associated with obesity in Tibetan adults. As Fig. 5 shows, after adjustment, the highest tertile group of GRS (≥ 3) and wGRS (≥ 0.55) were significantly associated with obesity in Tibetan adults.

We assessed the additive and multiplicative interactions between the *MC4R* gene polymorphisms with the consistently significant associated environmental factors (smoking, occupational physical activity, and leisure-time exercise) based on the dominant inheritance model. As the Supplementary Table S2 and Table S3 show, no significant interaction was found.

## Discussion

The Tibetan is a characteristic ethnic in western China, mainly settling in the Qinghai-Tibet Plateau at high altitudes. Up to now, the data on obesity in this population is limited. In this study, using a population-based cross-sectional survey, we reported the prevalence and associated factors of obesity and overweight in Tibetan adults in Qinghai, China. The results indicated that according to WGOC criteria, nearly half of the Tibetan adults were overweight or obesity. The age- and sex-standardized prevalence of obesity was about 18%. Besides the sex and age, the study showed that the modifiable factors (including smoking, alcohol consumption, physical activity, and exercise) were associated with obesity and overweight in this population. This study verified the contribution of single nucleotide polymorphisms of the *MC4R* gene to obesity, but a significant association was not found in *FTO*.

The prevalence of obesity in China is experiencing rapid growth in recent decades, rising from 7.1% to 2002 to 12% in 2010 [9]. According to the latest round of China National Nutrition Surveys (CNNSs) in 2015-19, 34.3% and 16.4% of adults ≥ 18 years in China were overweight and obesity [9]. Our study showed that the prevalence of overweight and obesity in Tibetan adults was near the average in China. The pattern of the obesity epidemic in China varies by geographic location and demographic clusters [25]. Obesity in different ethnicities differs because of the genetic ancestry, ecological environment, and importantly the diverse lifestyles. As a multi-ethnic cross-sectional study, the CNHS provides the opportunity of comparing the health status of ethnicities located in different regions in China. According to the published results of the CNHS, the obesity prevalence in Tibetan adults was higher than that in Bouyei in Guizhou [15], Yi in Sichuan [26], Uyghur in Xinjiang [13], and was slightly lower than that in Mongolian in Inner Mongolia [27] and Yugur in Gansu [28].

Obesity occurs with the combined contribution of genetic regulation and the obesogenic environment. While the fact is the worldwide epidemic of obesity is mostly attributed to the lifestyle transition as the human gene is unlikely to substantially change in the past few decades. It is the food environment and the environmentally driven reductions in physical activity that trigger the weight gain in genetically susceptible individuals [29]. We compared the obesity-related lifestyles in Tibetan and other ethnicities in China and found that Tibetan adults were more prevalent in light level physical activity than Bouyei [15] and Yi [26], which may partly explain the disparities in obesity. The leisure-time exercise in Tibetan was high but it was most likely the sequentially behavior change because of the presence of obesity [30]. We did not collect the dietary data in the study, previous studies identified the major dietary patterns in Tibetan adults - urban, western, and pastoral patterns [31]. Among these patterns, beef and mutton were the common sources of meat. The urban dietary pattern was characterized by frequent consumption of vegetables, tubers/roots, and refined carbohydrates. The western pattern was rich in sweetened drinks, snacks, and desserts, and the pastoral pattern featured tsamba (roasted Tibetan barley), Tibetan cheese, butter tea/milk tea, and whole-fat dairy foods. These dietary factors were found to be associated with an increased risk of metabolic syndrome, including obesity [31]. Besides, a proportion of Tibetan in China practice Tibetan Buddhism, which may affect the social practices and lifestyles, and may therefore have an impact on obesity. However, we did not collect information on religious beliefs. The interrelationship of religion, lifestyles, and health outcomes remain to be explored.

The contributions of genetic factors to body weight regulation have been proved. The heritability of BMI was estimated to range from 40 to 70% [32]. Association between SNPs in *FTO* and *MC4R* gene with obesity were widely replicated in different ethnic groups [33, 34]. Among Chinese, the associations have been demonstrated in Han people [35], but rarely in ethnic majorities. In this study, we designed the pair-matched case-control study based on the cross-sectional data and found that *MC4R* gene polymorphisms were determinants of obesity in Tibetan. Our previous work explored the same SNPs as this study in Yi people and showed similar results [36]. Notably, the minor allele frequencies (MAF) in Tibetan were higher than that in other ethnicities in China [36, 37], which provide a potential mechanism for the prevalence disparities. The gene-environment interaction is what we expect to discover, as it not only helps explain the underlying mechanism of disease occurrence but also provides a basis for eliminating environmental risk factors. However, in this study, neither the additive nor the multiplicative gene-environment interaction was found. It suggests that more genetic determinants and detailed environmental factors need to be further explored.

One of the strengths of this study lies in the collection of large-scale population-based data on the unique ethnic living at the high altitudes in China. It provides the diversity of the obesity epidemic in different ethnicities and geographic locations in China. Another strength is that this study comprehensively evaluated the environmental and genetic determinants of obesity in Tibetan.

One major limitation of this study is the lack of some important obesity-related variables, especially dietary data. Hence, unfortunately, we cannot analyze the impact of nutrition on obesity in Tibetan, nor adjust the confounding when analyzing other variables of interest. The future works are expected to explore the integrated factors associated with obesity in Tibetan. Secondly, we only used BMI to define obesity, which cannot distinguish fat and lean tissue or the body fat distribution. Further analysis is expected to define obesity by combining body muscle and body fat. Besides, obesity is a polygenic inheritable disease, while in this study we only detected four SNPs in two genes. The high throughput screening and verification are expected for a full understanding of the genetic mechanism of obesity in this population.

In conclusion, this study suggests that the prevalence of obesity and overweight in Tibetan adults was high. Both modifiable environmental factors and *MC4R* gene polymorphisms were associated with the obesity risk in this population. This study calls for the urgent need to improve education attainment especially health literacy. Healthy lifestyle adherence is also emphasized for obesity prevention.

### Electronic supplementary material

Below is the link to the electronic supplementary material.

Supplementary Material 1

## Data Availability

The datasets used and analysed during the current study are available from the corresponding author on reasonable request.
