# Supplementary material for: Prevalence, associated factors, and gene polymorphisms of obesity in Tibetan adults in Qinghai, China
Source: BMC Public Health. 2024 Jan 26;24:305. doi: 10.1186/s12889-023-17181-7 (PMC10811834; doi:10.1186/s12889-023-17181-7)
Supplement: Supplementary file 1 — Supplementary Material 1 [file 12889_2023_17181_MOESM1_ESM.docx]

Supplementary Table S1 Multivariable logistic analysis of obesity and overweight in Tibetan adults by deleting participants with BMI<18.5 kg/m^2^

| Variable | | Obesity | | Overweight | | |  |
| --- | --- | --- | --- | --- | --- | --- | --- |
|  |  | OR (95% CI) | P | | OR (95% CI) | P | |
| Sex, (Males, ref=Female) | | 2.30 (1.59, 3.34) | <0.0001 | | 1.81 (1.30, 2.50) | 0.0004 | |
| Age (years, reg=20-29) | |  |  | |  |  | |
|  | 30~39 | 3.89 (2.87, 5.28) | <0.0001 | | 2.36 (1.19, 4.66) | 0.0137 | |
|  | 40~49 | 8.11 (5.98, 10.99) | <0.0001 | | 3.69 (1.89, 7.20) | 0.0001 | |
|  | 50~59 | 10.42 (6.67, 16.28) | <0.0001 | | 4.30 (2.62, 7.08) | <0.0001 | |
|  | 60~80 | 8.55 (6.74, 10.84) | <0.0001 | | 2.70 (1.61, 4.53) | 0.0002 | |
| Residence (Rural, ref=Urban) | | 0.92 (0.76, 1.12) | 0.4103 | | 0.88 (0.76, 1.03) | 0.1029 | |
| Income (CNY/y, ref=<10,000) | | 0.87 (0.71, 1.07) | 0.1812 | | 1.23 (0.93, 1.63) | 0.1500 | |
| Education (Ref=Primary or below) | |  |  | |  |  | |
|  | Junior or senior high | 0.99 (0.59, 1.64) | 0.9594 | | 0.87 (0.69, 1.11) | 0.2756 | |
|  | College or above | 0.98 (0.57, 1.67) | 0.9284 | | 0.82 (0.54, 1.24) | 0.3370 | |
| Smoke (Ref=Never) | |  |  | |  |  | |
|  | Former | 0.83 (0.47, 1.45) | 0.5066 | | 0.79 (0.67, 0.93) | 0.0038 | |
|  | Current | 0.43 (0.32, 0.59) | <0.0001 | | 0.68 (0.47, 0.98) | 0.0367 | |
| Drink (Ref=Never) | |  |  | |  |  | |
|  | Former | 1.01 (0.55, 1.86) | 0.9695 | | 1.36 (1.06, 1.76) | 0.0170 | |
|  | Current | 0.84 (0.49, 1.45) | 0.5275 | | 0.98 (0.75, 1.28) | 0.8804 | |
| Occupational physical activity (Ref=Light) | | |  | |  |  | |
|  | Moderate | 0.90 (0.74, 1.09) | 0.2769 | | 0.75 (0.55, 1.04) | 0.0826 | |
|  | Heavy | 0.78 (0.67, 0.92) | 0.0029 | | 0.69 (0.58, 0.83) | <0.0001 | |
| Leisure-time exercise (Ref=Light) | | |  | |  |  | |
|  | Moderate | 1.22 (1.03, 1.46) | 0.0243 | | 0.95 (0.88, 1.02) | 0.1439 | |
|  | Heavy | 1.48 (1.07, 2.03) | 0.0170 | | 1.46 (1.18, 1.81) | 0.0005 | |

Supplementary Table S2 Additive interaction between *MC4R* gene polymorphism and environmental factors based on dominant inheritance model

| Gene | Environment | OR(95%CI) | P | RERI | AP | SI |
| --- | --- | --- | --- | --- | --- | --- |
| Rs17782313 | Smoking |  |  |  |  |  |
| T/T | Current | 1.00 | - |  |  |  |
| T/C+C/C | Current | 1.57 (0.65, 3.77) | 0.3136 |  |  |  |
| T/T | Never or former | 3.30 (1.53, 7.12) | 0.0023 |  |  |  |
| T/C+C/C | Never or former | 5.29 (2.39, 11.68) | <0.0001 | 1.41 (-4.18, 7.02) | 0.27 (-0.64, 1.18) | 1.49 (0.29, 7.56) |
| Rs12970134 | Smoking |  |  |  |  |  |
| G/G | Current | 1.00 | - |  |  |  |
| G/A+A/A | Current | 1.07 (0.45, 2.54) | 0.8745 |  |  |  |
| G/G | Never or former | 2.67 (1.30, 5.50) | 0.0075 |  |  |  |
| G/A+A/A | Never or former | 4.44 (2.07, 9.53) | 0.0001 | 1.69 (-2.63, 6.02) | 0.38 (-0.39, 1.15) | 1.97 (0.31, 12.28) |
| Rs17782313 | Occupational physical activity |  |  |  |  |  |
| T/T | Moderate or heavy | 1.00 | - |  |  |  |
| T/T | Light | 1.08 (0.61, 1.93) | 0.7735 |  |  |  |
| T/C+C/C | Moderate or heavy | 1.66 (0.86, 3.22) | 0.1332 |  |  |  |
| T/C+C/C | Light | 1.73 (0.98, 3.06) | 0.0602 | -0.02 (-1.83, 1.79) | -0.01 (-1.06, 1.04) | 0.97 (0.08, 11.19) |
| Rs12970134 | Occupational physical activity |  |  |  |  |  |
| G/G | Moderate or heavy | 1.00 | - |  |  |  |
| G/G | Light | 1.00 (0.58, 1.74) | 0.9875 |  |  |  |
| G/A+A/A | Moderate or heavy | 1.41 (0.71, 2.77) | 0.3249 |  |  |  |
| G/A+A/A | Light | 1.56 (0.90, 2.71) | 0.1113 | 0.15 (-1.41, 1.71) | 0.10 (-0.87, 1.07) | 1.37 (0.04, 46.40) |
| Rs17782313 | Leisure-time exercise |  |  |  |  |  |
| T/T | Light | 1.00 | - |  |  |  |
| T/C+C/C | Light | 1.79 (0.97, 3.31) | 0.0617 |  |  |  |
| T/T | Moderate or heavy | 1.92 (1.14, 3.22) | 0.0142 |  |  |  |
| T/C+C/C | Moderate or heavy | 2.89 (1.66, 5.04) | 0.0002 | 0.18 (-2.27, 2.63) | 0.06 (-0.76, 0.89) | 1.11 (0.28, 4.39) |
| Rs12970134 | Leisure-time exercise |  |  |  |  |  |
| G/G | Light | 1.00 | - |  |  |  |
| G/A+A/A | Light | 1.42 (0.78, 2.58) | 0.2572 |  |  |  |
| G/G | Moderate or heavy | 1.70 (1.04, 2.78) | 0.0359 |  |  |  |
| G/A+A/A | Moderate or heavy | 2.65 (1.53, 4.59) | 0.0005 | 0.57 (-1.55, 2.62) | 0.20 (-0.51,0.92) | 1.48 (0.30, 7.40) |

RERI: relative excess risk due to interaction. AP: attributable proportion due to interaction. SI: synergy index. Models were adjusted for residence, education, income, smoking status, drinking status, occupational physical activity, and leisure-time exercise.

Supplementary Table S3 Multiplicative interaction between *MC4R* gene polymorphism and environmental factors based on dominant inheritance model

|  | β | SE | OR (95%CI) | *P* |
| --- | --- | --- | --- | --- |
| Rs17782313 |  |  |  |  |
| T/C+C/C | 0.45 | 0.45 | 1.57 (0.65, 3.77) | 0.3136 |
| Never or former smoking | 1.19 | 0.39 | 3.30 (1.53, 7.12) | 0.0023 |
| (T/C+C/C) × Smoking | 0.02 | 0.49 | 1.02 (0.39, 2.69) | 0.9671 |
| Rs12970134 |  |  |  |  |
| G/A+A/A | 0.07 | 0.44 | 1.07 (0.45, 2.54) | 0.8745 |
| Never or former smoking | 0.98 | 0.37 | 2.67 (1.30, 5.50) | 0.0075 |
| (G/A+A/A) × Smoking | 0.44 | 0.49 | 1.55 (0.60, 4.02) | 0.3677 |
| Rs17782313 |  |  |  |  |
| T/C+C/C | 0.51 | 0.34 | 1.66 (0.86, 3.22) | 0.1332 |
| Light occupational physical activity | 0.08 | 0.29 | 1.08 (0.61, 1.93) | 0.7735 |
| (T/C+C/C) × Physical activity | -0.04 | 0.41 | 0.96 (0.43, 2.13) | 0.9139 |
| Rs12970134 |  |  |  |  |
| G/A+A/A | 0.34 | 0.35 | 1.41 (0.71, 2.77) | 0.3249 |
| Light occupational physical activity | 0.00 | 0.28 | 1.00 (0.58, 1.74) | 0.9875 |
| (G/A+A/A) × Physical activity | 0.10 | 0.42 | 1.11 (0.49, 2.52) | 0.8093 |
| Rs17782313 |  |  |  |  |
| T/C+C/C | 0.58 | 0.31 | 1.79 (0.97, 3.31) | 0.0617 |
| Moderate or heavy leisure-time exercise | 0.65 | 0.27 | 1.92 (1.14, 3.22) | 0.0142 |
| (T/C+C/C) × Exercise | -0.17 | 0.40 | 0.84 (0.39, 1.83) | 0.6646 |
| Rs12970134 |  |  |  |  |
| G/A+A/A | 0.35 | 0.31 | 1.42 (0.78, 2.58) | 0.2572 |
| Moderate or heavy leisure-time exercise | 0.53 | 0.25 | 1.70 (1.04, 2.78) | 0.0359 |
| (G/A+A/A) × Exercise | 0.10 | 0.38 | 1.10 (0.52, 2.34) | 0.7976 |

SE: standard error. Models were adjusted for residence, education, income, smoking status, drinking status, occupational physical activity, and leisure-time exercise.
